# A digital twin auxiliary approach based on adaptive sparse attention network for diesel engine fault diagnosis

**DOI:** 10.1038/s41598-021-04545-5

**Published:** 2022-01-13

**Authors:** Jiajie Jiang, Hui Li, Zhiwei Mao, Fengchun Liu, Jinjie Zhang, Zhinong Jiang, He Li

**Affiliations:** 1 Key Laboratory of Engine Health Monitoring-Control and Networking Ministry of Education, Beijing University of Chemical Technology, Beijing, 100029 China; 2 China North Engine Research Institute (Tianjin), Tianjin, 300400 China

**Keywords:** Mechanical engineering, Information theory and computation

## Abstract

Condition monitoring and fault diagnosis of diesel engines are of great significance for safety production and maintenance cost control. The digital twin method based on data-driven and physical model fusion has attracted more and more attention. However, the existing methods lack deeper integration and optimization facing complex physical systems. Most of the algorithms based on deep learning transform the data into the substitution of the physical model. The lack of interpretability of the deep learning diagnosis model limits its practical application. The attention mechanism is gradually developed to access interpretability. In this study, a digital twin auxiliary approach based on adaptive sparse attention network for diesel engine fault diagnosis is proposed with considering its signal characteristics of strong angle domain correlation and transient non-stationary, in which a new soft threshold filter is designed to draw more attention to multi decentralized local fault information dynamically in real time. Based on this attention mechanism, the distribution of fault information in the original signal can be better visualized to help explain the fault mechanism. The valve failure experiment on a diesel engine test rig is conducted, of which the results show that the proposed adaptive sparse attention mechanism model has better training efficiency and clearer interpretability on the premise of maintaining performance.

## Introduction

The diesel engine is an important power machine, which is widely used in ships, the military industry, nuclear power, and other fields. Because of its compact structure, complex motion, and poor working environment, the probability of failure is relatively high. If the equipment fails, it will often cause serious safety losses and a lot of maintenance costs^1^. Therefore, it is very necessary to study the health evaluation and fault diagnosis of diesel engines.

In the past decades, diagnosis methods based on mechanical vibration data have been widely used in the literature^2^. Traditionally, signal analysis and processing methods such as time-domain analysis^3^, frequency-domain analysis^4^, time–frequency analysis^5,6^ can be used to process vibration signals to achieve the purpose of fault diagnosis. However, the above methods rely heavily on fault mechanism and signal processing related professional knowledge and rich experience, which are difficult to deal with complex mechanical equipment systems under variable working conditions. The data-driven intelligent fault diagnosis method can not only effectively and quickly process mechanical signals, provide accurate diagnosis results, but also doesn’t need too much professional knowledge. Therefore, the deep learning algorithm, one of the most popular data-driven methods, has attracted more and more attention in the research of fault diagnosis^7–10^.

Although deep learning has achieved phased results in the field of fault diagnosis, data-driven methods rely on statistical models to determine the health status of the system. When the historical data is insufficient or the operating environment changes suddenly, it may not be enough to perform the health monitoring task. Therefore, the digital twin concept of data-driven and physical model integration has gradually attracted extensive attention in the industrial field^11–13^. Complex physical systems are often difficult to establish an accurate mathematical model, and it is impossible to evaluate their state and control optimization by analyzing. Digital twin uses data-driven methods to update, modify, connect and supplement the mathematical models by adding the historical and real-time operational data of the system. Integrating the system mechanism and operational data can better evaluate the system dynamically in real time. However, the existing methods lack deeper integration and optimization. Most of them convert the data into the substitution of physical models based on complex algorithms such as deep learning. The interpretability of the models is insufficient and it is difficult to deeply describe or characterize the mechanism of the system^14^. This greatly limits the application and development of the digital twin method in the industrial field. Therefore, some researchers try to add attention mechanisms to the network, exploring the relationship between the data in the model and the output process and basis of the network.

At present, attention mechanism has been widely and successfully applied to research tasks such as image caption generation^15,16^, document classification^17,18^, speech recognition^19,20^, text translation^21,22^. For example, Xu et al.^15^ revealed the corresponding relationship between words and images through the attention mechanism. Yang et al.^17^ proposed the hierarchical attention structure of 'word sentence article', finding the dependence on sentences and words in the text in the process of documents classification. Chan et al.^19^ illustrated the alignment process between audio signals and characters in the network by applying the attention mechanism. Vaswani et al.^21^ created the self-attention structure of named transformer, which visualizes the relationship between any two words of all in the text translation and clarifies the syntax of sentences. In the field of fault diagnosis, Li et al.^23^ assisted the deep network in locating effective information data segments by introducing an attention mechanism. Yang et al.^24^ employed a neural network model combining convolutional neural network, gated recursive unit, and attention mechanism, explaining the feature extraction process of the neural network on bearing data set. Zhao et al.^25^ generated a soft threshold by the attention mechanism in the deep network to filter information in the channel, which filters out the unimportant feature information and significantly improves the anti-noise performance. These deep networks can be integrated into digital twin systems to improve model reasoning and decision-making^26,27^.

Most of the above attention mechanisms can be classified as global attention mechanisms according to Ref.^28^. The global attention mechanism considers all sequences in the data. On the one hand, the network disperses the attention weight of each sequence and reduces the interpretability. On the other hand, it increases the computing cost and affects the computing efficiency of the network. Especially for long sequence data, it will significantly lead to the decline of network performance^29^. Accordingly, Luong et al.^28^ put forward the local attention mechanism. The network no longer pays attention to the global sequence, but only focuses on the nearby region of the target sequence, which has the lower computational cost. However, the local attention mechanism ignores the impact of non-adjacent sequences on the results, whose limitations are obvious^29^. Xue et al.^29^ further proposed the gated attention mechanism, in which the backbone attention network is a network containing the global attention mechanism. Then the binary gate is generated through the auxiliary network to dynamically select the concerned sequence into the backbone attention network. However, the auxiliary network in Ref.^29^ will expand the network scale to a certain extent. At the same time, there is also the problem of gradient disappearance for long sequences, which harms the generation of attention gating.

Due to the design of timing gear train and the existence of ignition impact, the signal of diesel engines has the characteristics of periodicity, transient and non-stationary^2^. The angular domain signal has a strong correlation with the crankshaft phase, resulting in a considerable part of the signal is redundant for fault diagnosis. Compared with the signals of gears and rolling bearings^2^, the key frequency information is difficult to capture, which brings difficulties to the further development of diesel engine fault diagnosis.

To solve the above problems, inspired by the idea of soft threshold filtering in Ref.^25^, this paper is no longer limited to the internal characteristics of the sequence but extends to sequences processing. Then build the binomial distribution function following the principle of backpropagation with the method in Ref.^30^. Further, propose A digital twin auxiliary approach named adaptive sparse attention network (ASAN). The network mainly makes use of the advantages of convolutional neural networks in complex signal feature extraction and the processing ability of bidirectional cyclic neural networks in sequence data, combined with an adaptive sparse attention module on this basis. The sparse attention module dynamically generates attention weights for each sequence data and adaptively calculates a soft threshold that is used to filter the sequence weights in real time. In this way, the reserved sequences are not limited to only one region but also fewer sequences are transferred to the next layer of the network, thus achieving the purpose of sparse attention. The proposed ASAN method reduces the computational cost of the global attention method and increases the training efficiency of the network, improving the network performance compared with the local attention method. At the same time, this method also has better interpretability, which can further explore the interval of fault signal characteristics and study the fault signal mechanism. In the face of other faults, the model can be further updated and optimized^31^ according to the real-time data.

The main contributions of this paper are as follows:

1. A new digital twin auxiliary approach named adaptive sparse attention network (ASAN) is proposed, which uses a soft threshold to dynamically select the data sequences that need attention in real time. It avoids iterative calculation of redundant sequences and reduces the calculation cost of the network, allowing the model to focus on more important sequences in the data and improves the interpretability.
2. The effects of independent and shared convolution parameters on the network are compared. It is found that the convolution parameters of each sequence independently set in three different input modes (angular domain, frequency domain, envelope spectrum) can significantly improve the performance of the model and enhance the generalization ability of the network.
3. Fault simulation experiments including different valve conditions under three loads are carried out on a 12 cylinder diesel engine in the laboratory, and the effectiveness of the proposed algorithm is verified. On the premise of maintaining the performance of the model, the proposed algorithm has better training efficiency and interpretability, which can locate the range of fault characteristics and reveal the expression of signal characteristics. Then it lays a foundation for fault feature extraction and fault diagnosis in the next step.

## Proposed model (ASAN)

To locate the information segments closely related to different states of the diesel engine, the attention mechanism is used. Considering the sequence feature redundancy of diesel engine signal, an adaptive sparse attention model is further proposed. In addition, we visually display the attention weight distribution of the input samples to explore whether the decision-making process of the model is consistent with human experience. The proposed model and fault diagnosis process of digital twin is shown in Fig. 3.

### Convolution layer

Convolutional neural networks (CNNs) are specially designed to deal with complex signals. In the past few years, a large number of studies^32,33^ have been based on the characteristics of CNN's local receptive field, weight sharing, and spatial subsampling, so that the data information has the ability not to be affected by scale, displacement, and distortion. In this study, 1DCNN is used to extract the features of vibration signals.

Firstly, segment the data. Suppose that the input sequence data after segmentation is ${\text{x}} = \left[ {x_{1} ,x_{2} , \ldots ,x_{N} } \right]$, where N is the length of the sequence, that is, the number of segments. The convolution operation on each sequence can be defined as follows:

1 $$z_{i} = \varphi \left( {{\text{w}}x_{i} + b} \right),$$

where $b$ and $\varphi$ represent bias term and nonlinear activation function respectively. The output $z_{i}$ of the convolution layer is obtained by sliding the convolution kernel ${\text{w}}$ from the first point to the last point of $x_{i}$, which can be regarded as the feature learned by the convolution kernel on the corresponding sequence $x_{i}$. In addition, multiple convolution kernel stacking operations with different lengths can be applied in the convolution layer.

After that, the pooling layer needs to be applied to the output features generated by the convolution layer. On the one hand, pooling can extract the most important local information in each feature map. On the other hand, this operation can significantly reduce the feature dimension. In this paper, the maximum pool function is used. Each sequence pooling operation can be represented as follows:

2 $${\text{p}}_{i} { = }\left[ {p_{i}^{1} ,p_{i}^{2} , \ldots ,p_{i}^{s} } \right],$$

3 $$p_{i}^{k} = \max \left( {z_{i}^{{\left( {k - 1} \right)g + 1}} ,z_{i}^{{\left( {k - 1} \right)g + 2}} , \ldots ,z_{i}^{kg} } \right),$$

where g is the length of the pooled window, ${\text{p}}_{i}$ represents the output of the ith sequence feature after the pooled operation, and s represents the dimension. Multiple convolutions and pooling operations improve the learning ability of CNNs. At the same time, the network structure of multiple CNN layers can extract the low-dimensional and high-dimensional features of the original data. Reasonably setting parameters of CNN networks can make it better learn fault diagnosis knowledge.

### Bidirectional LSTM layer

The long short-term memory architecture (LSTM) can prevent the gradient disappearance of backpropagation to a certain extent, which is often used to model the dependence between long sequences^34^.

**Figure 1:**
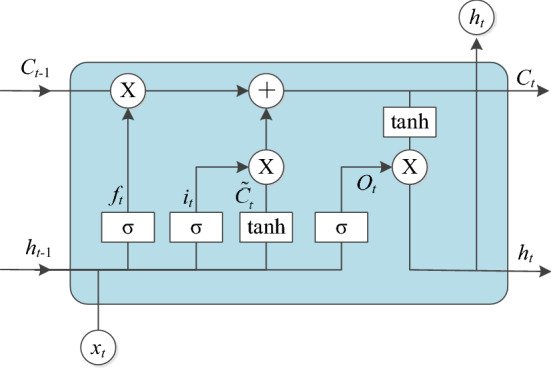
LSTM memory unit.

Figure 1 illustrates the calculation flow of an LSTM memory unit. At each time step, the computing unit is cut, written, and cleared through several gates to control the transmission of information along the data sequence, which enhances the learning ability of the model to capture the characteristics of long sequences. When a new input enters the unit, if the input door is opened, its information will accumulate to the unit. If the forgetting gate $f_{t}$ is activated, the output state $c_{t - 1}$ of the previous computing unit will be forgotten. The output gate $o_{t}$ controls whether the latest cell output $c_{t}$ is transferred to the hidden layer state $h_{t}$. In this study, the mainstream LSTM algorithm in Ref.^35^ is adopted, and the calculation in the unit is as follows:

4 $$i_{t} = \sigma \left( {w_{xi} x_{t} + w_{hi} h_{t - 1} + w_{ci} c_{t - 1} + b_{i} } \right),$$

5 $$f_{t} = \sigma \left( {w_{xf} x_{t} + w_{hf} h_{t - 1} + w_{cf} c_{t - 1} + b_{f} } \right),$$

6 $$c_{t} = f_{t} c_{t - 1} + i_{t} \tanh \left( {w_{xc} x_{t} + w_{hc} h_{t - 1} + b_{c} } \right),$$

7 $$o_{t} = \sigma \left( {w_{xo} x_{t} + w_{ho} h_{t - 1} + w_{co} c_{t} + b_{o} } \right),$$

8 $$h_{t} = o_{t} \tanh \left( {c_{t} } \right),$$

where $\sigma$ is the sigmoid activation function, $w_{hi}$ represents the transformation matrix from the hidden layer to the input gate, $w_{xc}$ represents the transformation matrix from the input state to the output gate. Accordingly, other subscripts and so on.

Although LSTM solves the problem of the disappearance of the sequence gradient to some extent, it can not be avoided completely, which leads to more attention to the sequence information behind it. Therefore, if LSTM can pay attention to both front and back sequences, the neural network is expected to obtain a better understanding of signals.

Bidirectional LSTM can solve the above problem by dividing the hidden layer into two parts: forward hidden sequence $\overrightarrow {h}$ and backward hidden sequence $\overleftarrow {h}$. The output layer can be updated through the following iterative process:

9 $$\overrightarrow {h}_{t} = \gamma \left( {w_{{x\overrightarrow {h} }} x_{t} + w_{{\overrightarrow {h} \overrightarrow {h} }} \overrightarrow {h}_{t - 1} + b_{{\overrightarrow {h} }} } \right),$$

10 $$\overleftarrow {h}_{t} = \gamma \left( {w_{{x\overleftarrow {h} }} x_{t} + w_{{\overleftarrow {h} \overleftarrow {h} }} \overleftarrow {h}_{t - 1} + b_{{\overleftarrow {h} }} } \right),$$

11 $$y_{t} = w_{{\overrightarrow {h} y}} \overrightarrow {h} + w_{{\overleftarrow {h} y}} \overleftarrow {h}_{t} + b_{y} .$$

### Adaptive sparse attention layer

This section details the structure of the adaptive sparse attention module and the source of the method. The feasibility of backpropagation is simply proved.

#### Classic attention mechanism

The attention mechanism is inspired by the brain's ability to solve overload information. In recent years, this method has been successfully applied in a wide range of tasks.

In order to locate the data information, the original input ${\text{x}}$ is divided into N segments. Then the high-dimensional feature $r$ extracted by the network is used as the input of the attention module. The vector $r_{i}$ is a high-dimensional representation of the ith segment.

The attention mechanism generates a positive weight $\alpha_{i}$ for $r_{i}$, which represents the importance of the corresponding data segment in the generation of the final result. $\alpha_{i}$ can be calculated by an attention model $f_{att}$ with $r_{i}$ as input and a softmax function.

12 $$u_{i} = f_{att} \left( {r_{i} } \right),$$

13 $$\alpha_{i} = \frac{{e^{{u_{i} }} }}{{\Sigma_{k = 1}^{{N_{seg} }} e^{{u_{k} }} }}.$$

$f_{att}$ can be a simple attention model, that is, a layer of neural network. When the attention weight of each segment is generated, the enhanced representation vector $v$ of the whole input data can be obtained:

14 $$v = \sum\limits_{i = 1}^{{N_{seg} }} {\alpha_{i} r_{i} } .$$

Then, you can use $v$ as a high-level representation of ${\text{x}}$ for further diagnosis. The network output using the softmax regression function is interpreted as the probability of each category, which is the result of the final fault classification diagnosis.

15 $$y = {\text{softmax}}\left( {W_{f} v + b_{f} } \right),$$

where $W_{f}$ and $b_{f}$ are the corresponding weight matrix and bias term respectively. It should be pointed out that the mechanism here is a typical method for learning the attention of neural networks. Similar attention methods are often used in other research tasks^23^.

#### Adaptive sparse attention structure

**Figure 2:**
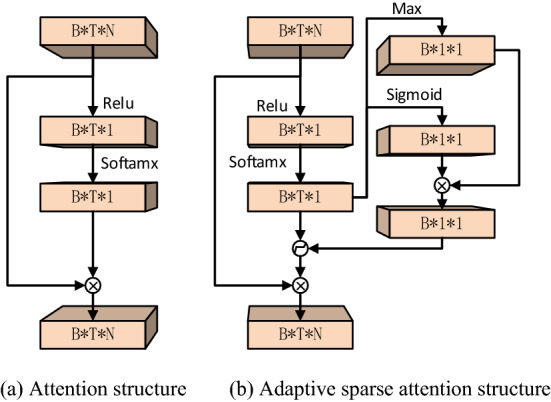
The change of adaptive sparse attention structure.

The proposed adaptive sparse attention is a variant of attention, which uses a soft threshold to remove unimportant sequence features. The soft threshold is inserted into the network structure as a nonlinear conversion layer. In addition, the threshold can be learned adaptively in the network. The specific process is described as follows:

As shown in Fig. 2b, this is an adaptive sparse attention structure. Different from the typical attention structure in Fig. 2a, it has a special module for estimating the threshold, in which the sigmoid function is used for the attention layer to scale the scaling parameters to the range of (0, 1), so as to obtain a preliminary threshold. The scaling process can be expressed as follows:

16 $$\alpha = {\text{sigmoid}}\left( {W_{\alpha } z + b_{\alpha } } \right),$$

where $z$ is the output of the attention layer in the network, which is a one-dimensional vector. $\alpha$ is the corresponding scaling parameter. Then, the scale parameter $\alpha$ is multiplied by the maximum value of the attention layer to the threshold. This arrangement takes into account that the threshold not only needs to be positive but also can not be too large. If the threshold is greater than the maximum absolute value of the attention layer, the output of the adaptive sparse attention is zero. To sum up, the threshold used by adaptive sparse attention is expressed as follows:

17 $$\tau = \alpha \cdot \max \left( z \right),$$

where $\tau$ is the final threshold. In this way, the threshold can be kept within a reasonable range, and the output of the adaptive sparse attention will not be all zero.

Then, the output of the attention layer is filtered through the threshold. This study refers to Ref.^30^ to construct the mask function for filtering. The sigmoid function with simple structure and easy transformation is selected. The mask function is as follows:

18 $$f\left( z \right) = relu\left( {\frac{1}{{b + e^{ - a(z - \tau )} }} - b} \right),$$

where $relu$ is a commonly used activation function, which directly sets the negative number to zero and retains the positive number. If $a$ is large enough, $b$ takes 0.618. When $\left( {z - \tau } \right) > 0$, the output result of $f\left( z \right)$ approaches 1. When $\left( {z - \tau } \right){ = 0}$, the output result of $f\left( z \right)$ approaches 0 but is greater than 0. This can prevent the gradient disappearance caused by $relu$. When $\left( {z - \tau } \right) < 0$, the output result of $f\left( z \right)$ is equal to 0. In this study, $a$ was set to 50.

**Figure 3:**
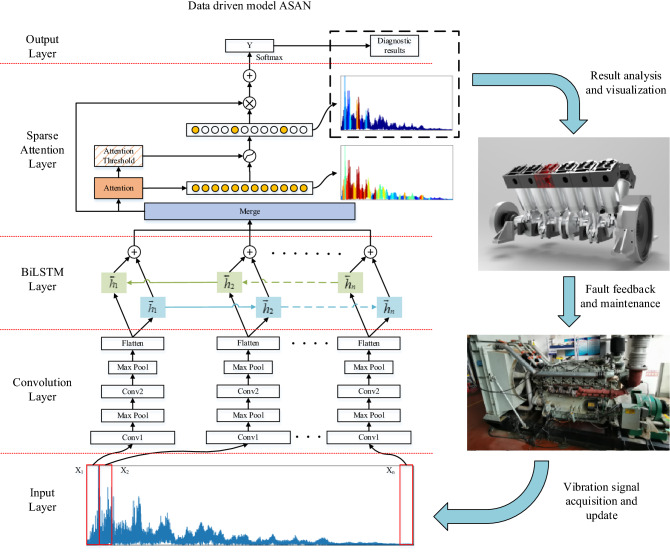
Fault diagnosis process of digital twin based on adaptive sparse attention network.

Finally, the filtered output and the output of the attention layer are recalculated by the softmax function to reconstruct the attention output. The calculation method is shown in the following formula:

19 $$newz_{i} = \frac{{f\left( {z_{i} } \right)e^{{z_{i} }} }}{{\Sigma_{i = 1}^{{N_{seg} }} f\left( {z_{i} } \right)e^{{z_{i} }} }},$$

where $N_{seg}$ is the number of sequences and $newz_{i}$ is the output of the sparse attention module (Fig. 3).

At the same time, this method conforms to the theory of reverse propagation. First, we can simplify the derivative equation as follows:

20 $$\frac{\partial loss}{{\partial \tau }} \propto \frac{\partial f}{{\partial \tau }}.$$

$loss^{\prime}\left( \tau \right)$ is proportional to $f^{\prime}\left( \tau \right)$. In optimization problems, $\tau$ increases if $loss^{\prime}\left( \tau \right) < 0$ and decreases otherwise. $f^{\prime}\left( \tau \right)$ represents the partial derivative of the mask function to $\tau$, which can be expressed by piecewise function:

21 $$f^{\prime}\left( \tau \right) = \left\{ {\begin{array}{*{20}l} { < 0} \hfill & {z \to \tau } \hfill \\ { = 0} \hfill & {otherwise.} \hfill \\ \end{array} } \right.$$

When $\tau$ approaches $z$, $f^{\prime}\left( \tau \right) < 0$. According to Eq. (20), $loss^{\prime}\left( \tau \right) < 0$, then $\tau$ will iterate in the increasing direction. If $\tau$ is given a small initialization and the distribution of $z$ is limited, as $\tau$ increases away from the smaller value and maintains a certain distance from the larger value, $f^{\prime}\left( \tau \right){ = }0$ and $loss^{\prime}\left( \tau \right){ = }0$, $\tau$ will not change. And its iterative process will be temporarily stopped. The case of $f^{\prime}\left( \tau \right) > 0$ is not considered here, because when $\tau$ is reduced, the filtered components in $z$ are reduced and the sequence entering the next step is increased, which is contrary to the original intention of the proposed algorithm (filtering sparseness as much as possible).

So, we constrained the iterative direction of $\tau$, either increasing or stopping. At the same time, in order to limit the distribution of $z$ and obtain reasonable results, the loss function is reconstructed as follows^30^:

22 $$L\left( X \right) = \mathop \Sigma \limits_{s = 1}^{2} L_{cls} \left( {Y^{\left( s \right)} ,Y^{*} } \right) + max\left\{ {0,p_{t}^{\left( 1 \right)} - p_{t}^{\left( 2 \right)} + margin} \right\},$$

where $s$ represents the output of each attention layer, $Y^{\left( s \right)}$ and $Y^{*}$ respectively represent the predicted label vector and the real label vector from specific attention layers. $L_{cls}$ represents the cross-entropy loss of classification to ensure the accuracy of each layer's attention output. In addition, the latter part of Eq. (22) is sorting loss, where $p_{t}^{\left( s \right)}$ represents the prediction probability of the network corresponding layer on the correct category label $t$. And $p_{t}^{\left( 1 \right)} + margin \le p_{t}^{\left( 2 \right)}$ is forced in training. This design ensures that the soft threshold filtering operation makes the network iterate in a better direction, which makes the generation of sparse attention more convincing. $margin$ takes 0.05 here.

The proposed adaptive sparse attention method will generate a soft threshold to obtain attention filtered output. The threshold is learned automatically in the depth architecture, not manually set by experts. Therefore, this method does not need the professional knowledge of signal processing, which has a certain positive significance in the face of difficult fault diagnosis and interpretability.

Relevant parameters of the proposed network are shown in Table 1.

**Table 1:** Network parameter settings.

Parameter	Value
Training epochs	300
Learning rate	0.001
Conv1 kernel size	8
Conv1 kernel length	3
Conv2 kernel size	16
Conv2 kernel length	3
LSTM hidden units	16

## Experimental study

### Experimental device and scheme

**Figure 4:**
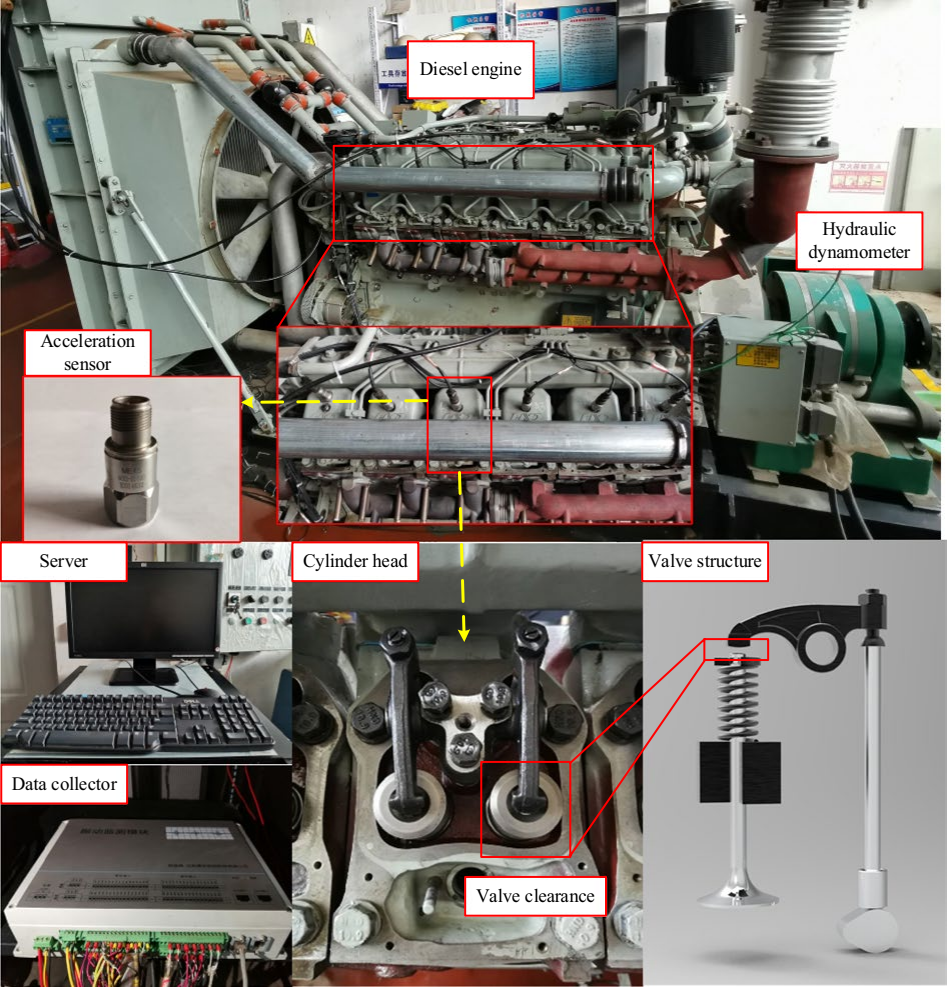
Layout of fault simulation test bench and sensor.

The test bench is a TBD234V12 direct injection diesel engine, whose key parameters are shown in Table 2. Vibration acceleration sensors are installed on the cylinder head of each cylinder to obtain its vibration signal. Eddy current sensors are installed in the radial and axial directions of the flywheel connected to the crankshaft to obtain the instantaneous speed signal and key signal respectively. The test data is collected through the BH5000E monitoring system. The main structure and layout of the test bench is shown in Fig. 4. The parameters of the acceleration sensor are shown in Table 3. The cylinder head vibration acceleration sensor is connected with the base by double studs, and the base is connected with the cylinder head surface by the adhesive. The data collector of the BH5000E has a 24-bit analog-to-digital converter (ADC) resolution, a maximum sampling rate of 102.4 KS/s per channel and 32 analog input ports. The collected signals are processed by a server with 16 GB RAM and a 3.10 GHz Intel i7 processor.

**Table 2:** Key parameters of TBD234V12 diesel engine.

Number of cylinders	Compression ratio	Idling/rpm	Structural style	Firing order
12	15:1	650	V type 60°	B1-A1-B5-A5-B3-A3-B6-A6-B2-A2-B4-A4

**Table 3:** Parameters of the accelerometer.

Performance specification	Unit	Accelerometer
Voltage sensitivity	mV/g	10
Frequency range (± 5%)	Hz	1–10,000
Frequency range (± 10%)	Hz	0.4–16,000
Natural frequency	Hz	42,000
Amplitude range	± g pk	500
Residual noise	g rms	0.0003
Mechanical shock limit	± g pk	5000
Temperature range	°C	− 55 to 125
Amplitude linearity	%	± 1

As one of the main motion mechanisms, the valve train of diesel engine often has the fault from abnormal increase of valve clearance due to wear and other reasons, which results in economic losses. The test takes the abnormal inlet and exhaust valve clearance as the target fault. Under the cold state of the unit, the exhaust valve clearance of cylinder A4 is changed by adjusting the bolt. The clearance is set quantitatively with the help of the feeler gauge. And the test is carried out under different working conditions.

### Data description

Vibration data of valves in different conditions under three loads were collected during the test. There were 6 working conditions in total. The detailed working condition settings are shown in Table 4. The speed of the diesel engine is 1500 rpm and the sampling frequency is 51,200 Hz. The TBD234V12 diesel engine is a four-stroke diesel engine. The crankshaft rotates for 2 cycles, that is, 720° is a period. Therefore, the single-period data points of the test diesel engine should be 51,200/(1500/60) × 2 = 4096. In this paper, samples with different numbers of periods are selected according to different input signals, which are normalized. The training set, verification set, and test set are divided according to the ratio of 6:2:2.

**Table 4:** Setting of experimental conditions.

Engine speed/rpm	Load/N m	Exhaust valve clearance/mm	Experimental marking
1500	0	0.5	N0
		0.9	F0
	400	0.5	N400
		0.9	F400
	800	0.5	N800
		0.9	F800

**Figure 5:**
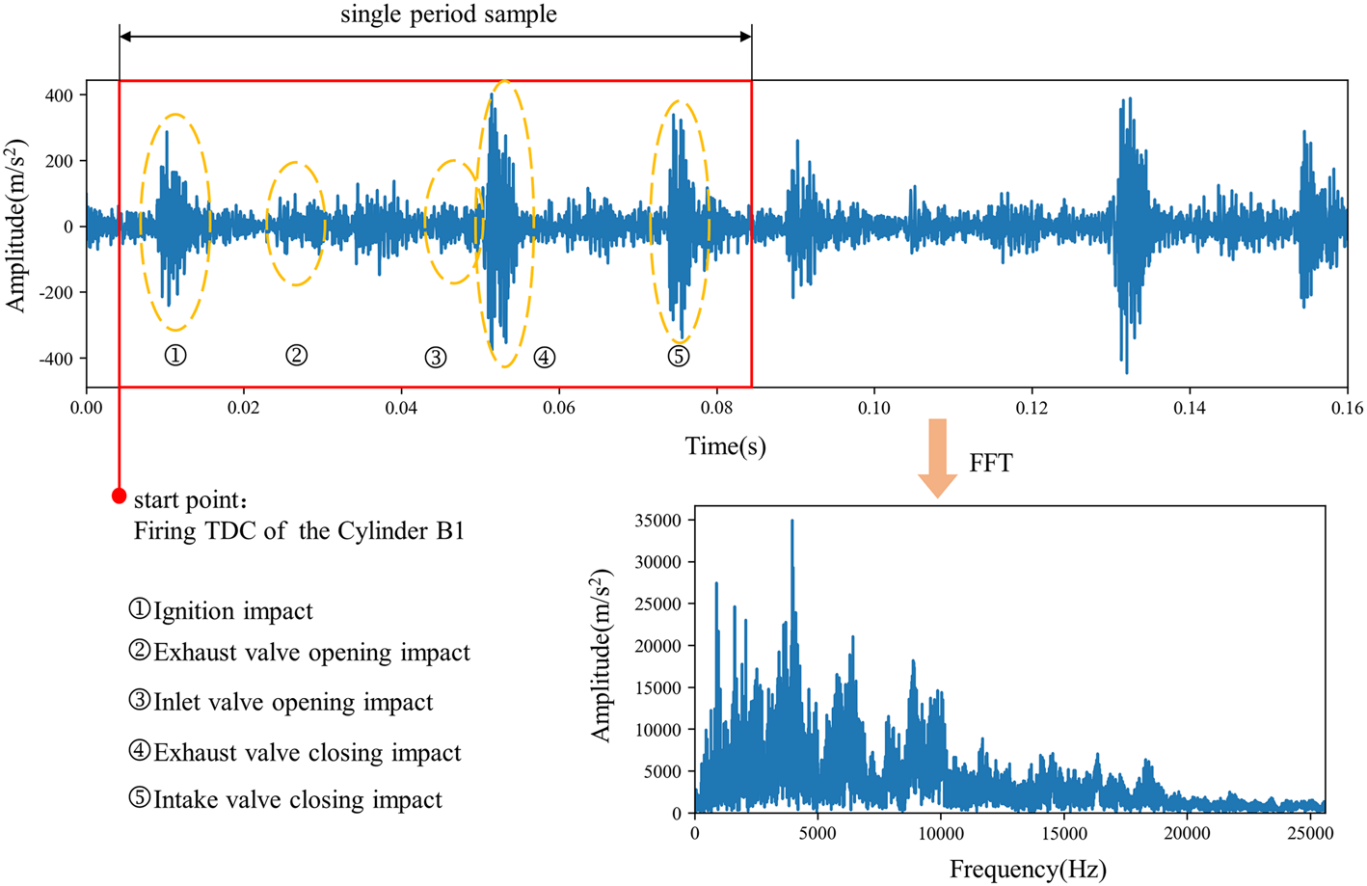
Acquired time and frequency domain signals.

The collected time-domain signal is shown in Fig. 5. Taking the vibration signal of cylinder A1 as an example, the impact closely related to cylinder action is ignition, exhaust valve opening, intake valve opening, exhaust valve closing and intake valve closing in turn. The starting point (0°) of each cylinder signal period is the firing TDC of cylinder B1. It is inferred that the firing phases of each cylinder are distributed according to the firing order spacing of 60°. The corresponding frequency-domain signal is shown in the figure, which is obtained by the Fourier transform directly from the time-domain signal in multiple periods.

Since the fault is only set in single cylinder A4, other cylinders are normal and the signal difference is weak. It is a more difficult task to diagnose the valve failure of the diesel through other normal cylinders. Therefore, the dataset contains a total of 6 cylinders from A1 to A6 to enhance the difficulty of fault diagnosis and classification. In addition, because the ignition phase of each cylinder in the operation of the diesel engine is different, it can not meet the input form requirements of the model. So the ignition phase of each cylinder is uniformly adjusted to align with the ignition of cylinder A1 in the data set, that is, the phase of firing TDC is near 60° relative to the firing TDC of cylinder B1 (0°). This paper mainly studies the fault of the exhaust valve, so the valve in the following text refers to the exhaust valve if there is no special description.

### Comparison of input methods

#### Angular domain

Firstly, the collected original mechanical vibration signal is directly converted to the angular domain as the model input to test the effect of the model in the angular domain. The time-domain signal is converted into the angular domain through instantaneous speed. The process is as follows:

23 $$angle = \int_{0}^{t} {\omega_{ins} } dt,$$

where, $\omega_{ins}$ is the instantaneous speed converted by the tooth spacing of the gear, and the integral of the instantaneous speed to time is the crankshaft angle $angle$ at the current time.

In the diesel engine angular domain data set, single-cylinder A4 data that is a period sample is used as input. The input points are 4096 and marked data $N_{train} = 30$ for training. Due to the design of the timing gear train, the cylinder head vibration has a strong correlation with the crankshaft angle. Although the signal is non-stationary in the time domain, it is invariant in the angle domain, which lays a foundation for the generalization of the model signal segmentation. At the same time, further considering the period of angle domain waveform, to avoid obvious impact waveform segmentation of the signal, the data is adjusted as follows as a preprocessing method:

24 $$\hat{x} = \left| {H\left( x \right)} \right|,$$

25 $$s = slide\left( {\hat{x}} \right),$$

26 $$\min \left\{ {\sum {\left[ {t^{i} - \left( {n^{i} + \theta } \right)} \right]}^{2} } \right\},$$

where $x$ is the original signal, $H$ is the Hilbert transform, $\hat{x}$ is the module length of the original signal after Hilbert transform. $\hat{x}$ obtains the signal $s$ through the smoothing function $slide$, and $t^{i}$ is the abscissa phase corresponding to the maximum point of $s$. $n^{i}$ is the center phase of the segmented region closest to $t^{i}$. $\theta$ is the initial phase of the angular domain signal. When the distance between $t^{i}$ and $n^{i} + \theta$ is the smallest, It can be considered that the signal adjustment is appropriate. Here, when the number of segments $n$ is 16, the initial phase $\theta$ is − 10°. It is generally believed that the features are hidden in the impact. Retaining the complete impact waveform is more conducive to the feature extraction of the model.

**Figure 6:**
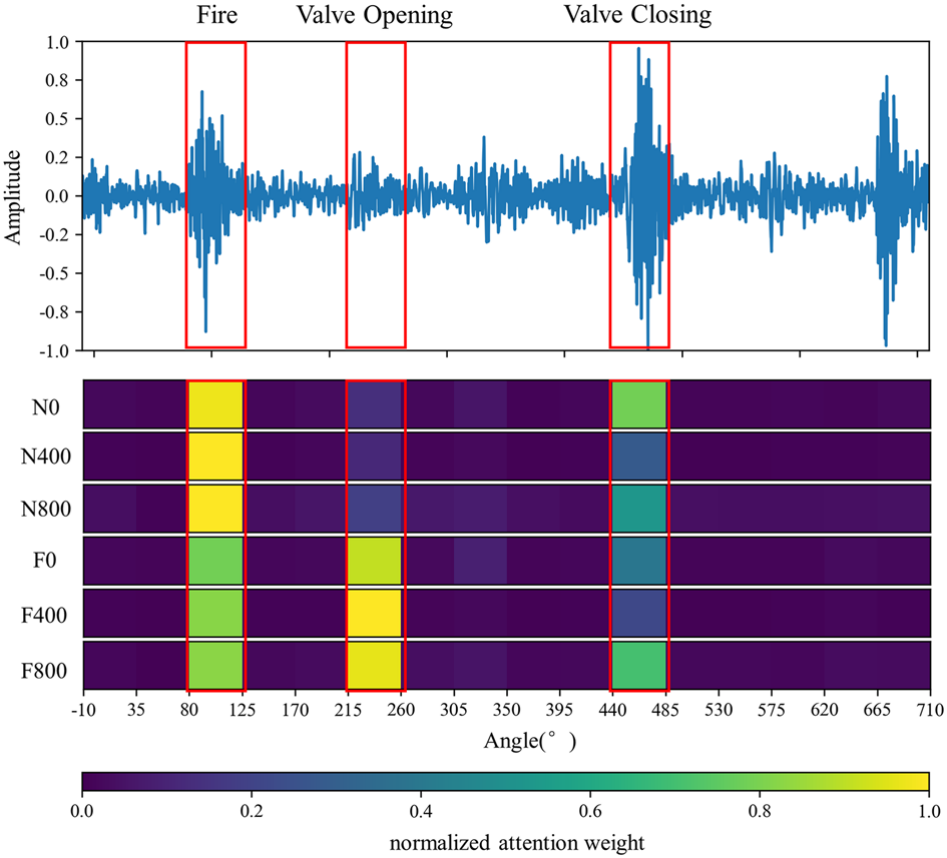
Attention for each working condition on angular domain.

**Figure 7:**
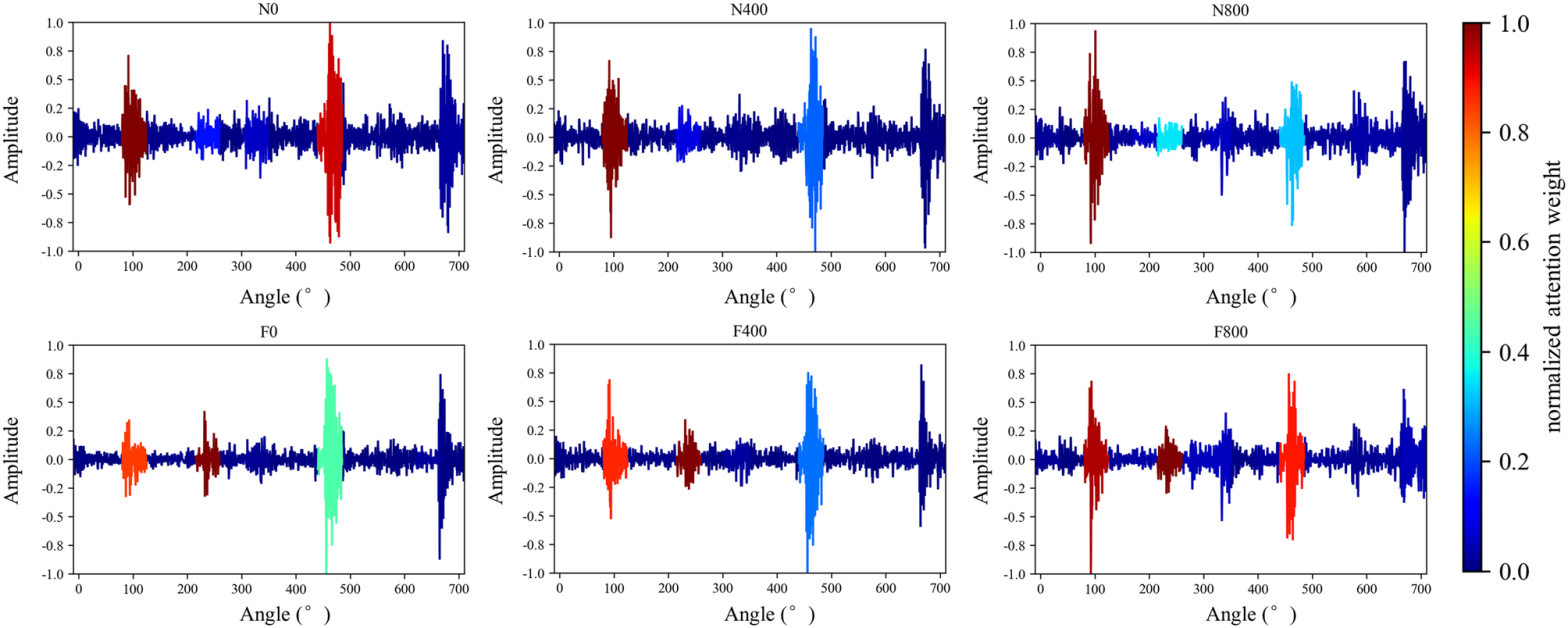
Visualization of attention weight on angular domain signal of various working conditions.

**Figure 8:**
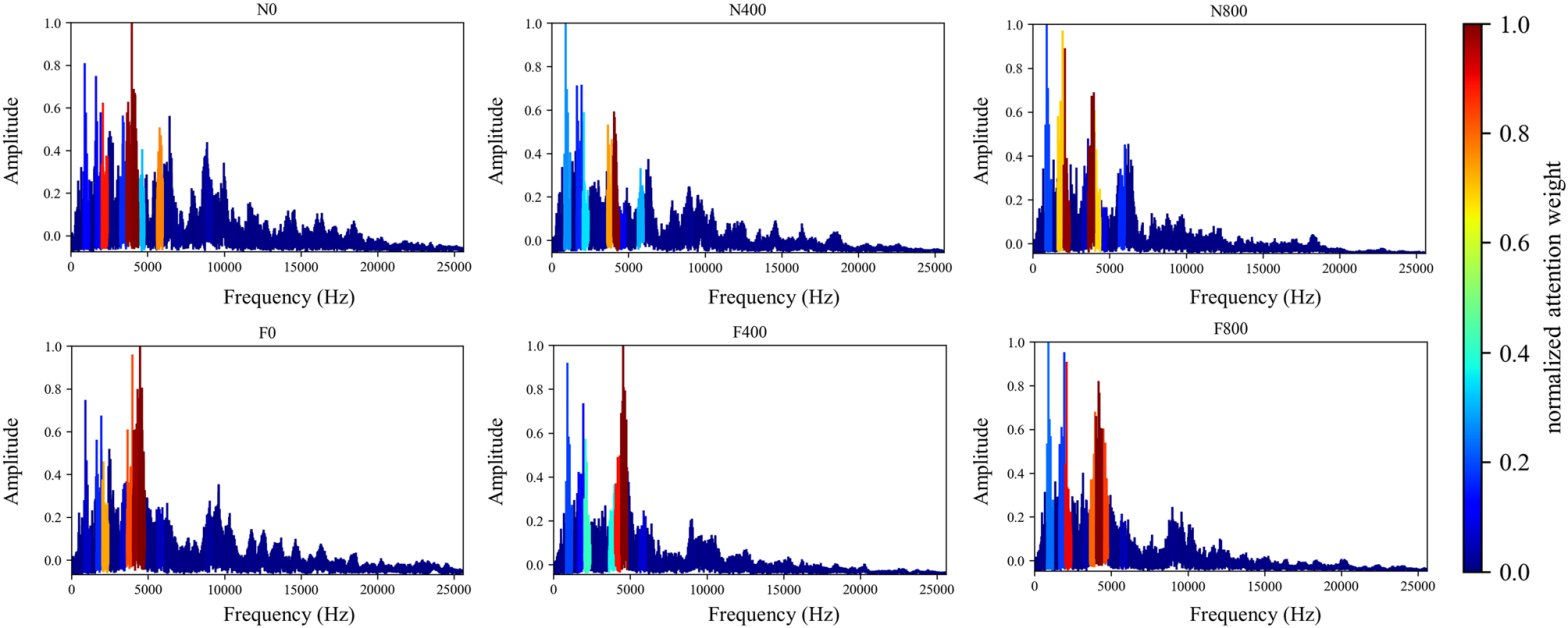
Visualization of attention weight on frequency domain signal of various working conditions.

**Figure 9:**
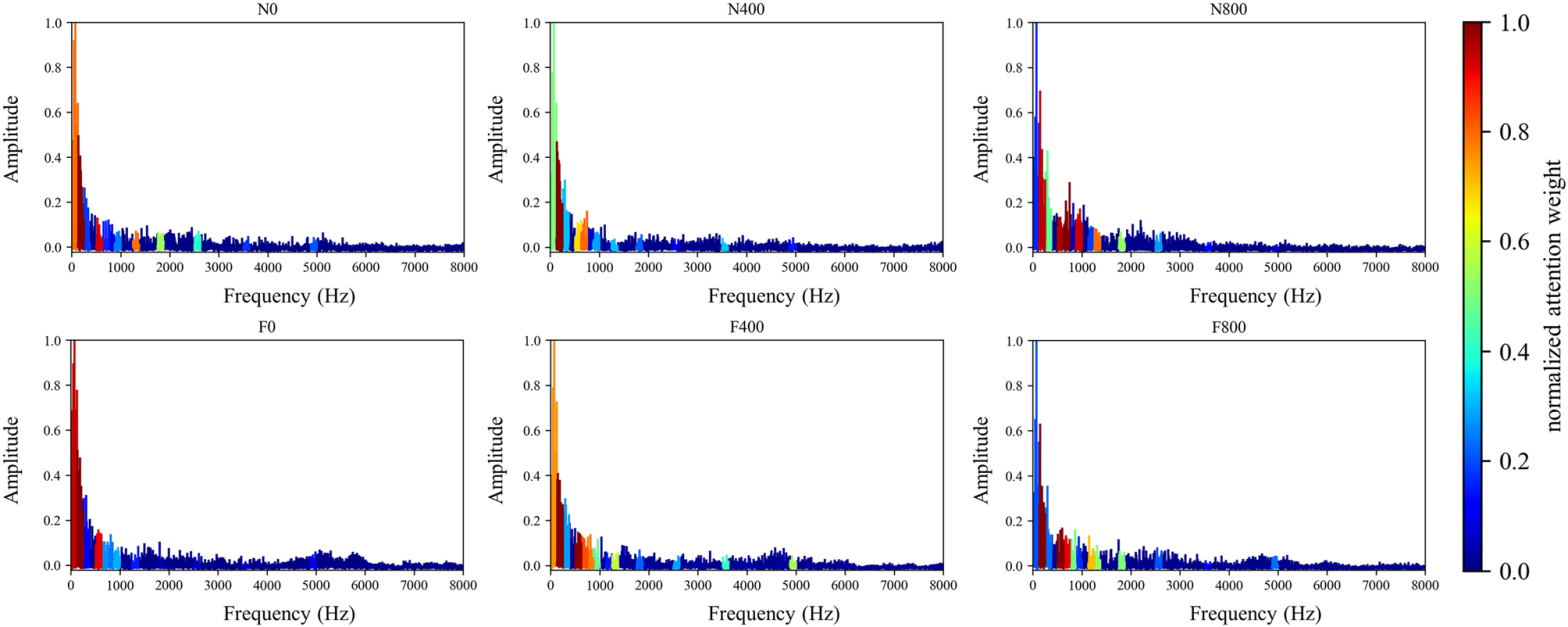
Visualization of attention weight on envelope spectrum signal (8000 Hz) of various working conditions.

The mechanical vibration signal of diesel engines' valve failure and different loads are intuitive. Generally speaking, the obvious change of valve impact amplitude and phase indicates that the valve fails, and the ignition impact of diesel engines will increase with the increase of load. Figure 6 shows the normalized average value of attention weight learned by the model for each working condition sample of the diesel engine data set. Figure 7 shows the visual attention weight of each working condition sample, where color indicates the size of attention weight and red represents the high level (Figs. 8, 9).

**Figure 10:**
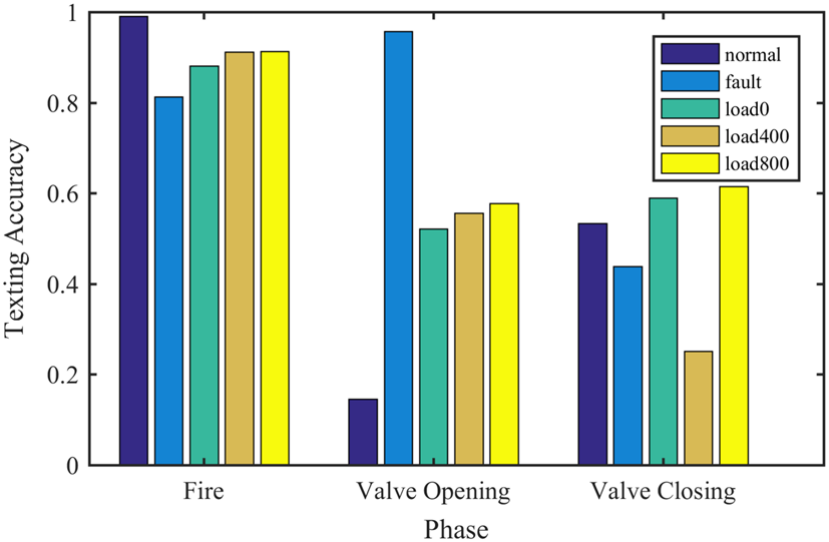
Attention weight summarized in the working condition.

It can be observed from Figs. 6 and 7 that the network mainly captures the peaks of angular domain signals, but not all peaks. Under rev1500load0, the attention peak of normal data is in the valve closing and ignition phase, and fault data’s is in valve opening and ignition phase; Under rev1500load400, the attention peak of normal data is in the ignition phase, and fault data’s is still in the valve opening and ignition phase; Under rev1500load800, the attention peak of normal data is in the firing phase, and the fault data has a large attention weight for multiple phases. In order to more accurately explain the variation rule between each characteristic phase and working conditions, this paper classifies the attention weight of each working condition sample and makes summary statistics according to Fig. 10.

When the diesel engine has abnormal exhaust valve clearance, the attention weight of ignition and exhaust closing decreases, and the attention weight on exhaust opening increases significantly. The intake and exhaust valves are opened and closed in a fixed phase. When the valve clearance increases abnormally, the collision wear between the rocker arm and the valve will become larger, which directly leads to the shortening of the valve opening process (i.e. the valve opening phase lags and the closing phase advances). The existence of the clearance will also lead to the obvious increase of the valve opening impact. In addition, the ignition of the diesel engine faulty cylinder will be negatively affected to a certain extent. The results of network attention are consistent with human experience. When the load of the diesel engine increases, the attention on the ignition and valve opening of cylinder A4 have a certain growth trend, and the valve closing impact also fluctuates greatly, which is consistent with manual experience.

The network can identify the direct feature of the valve fault, that is, the valve phase, and the indirect feature, that is, the ignition-related phase. In addition, with the increase of load, the ignition working condition of the diesel engine will change. The network can also identify ignition and other related phases adaptively according to the load. This shows that the deep network structure can learn the discrimination characteristics of the diesel engine under different working conditions, which are consistent with the basic rule of human fault diagnosis knowledge.

#### Frequency domain

In this section, the effect of the attention mechanism in the spectrum that is used as the model input is studied. Similar to the vibration angle domain signal, the spectrum also provides an intuitive view for fault diagnosis, especially fault location and identification. Figure 8 shows the attention visualization learned on the diesel spectrum dataset. Single-cylinder A4 data that is four periods samples are used as input. The number of input points is 16,384, the labeled data $N_{train} = 30$ is used for training, and the number of segments is 64.

**Figure 11:**
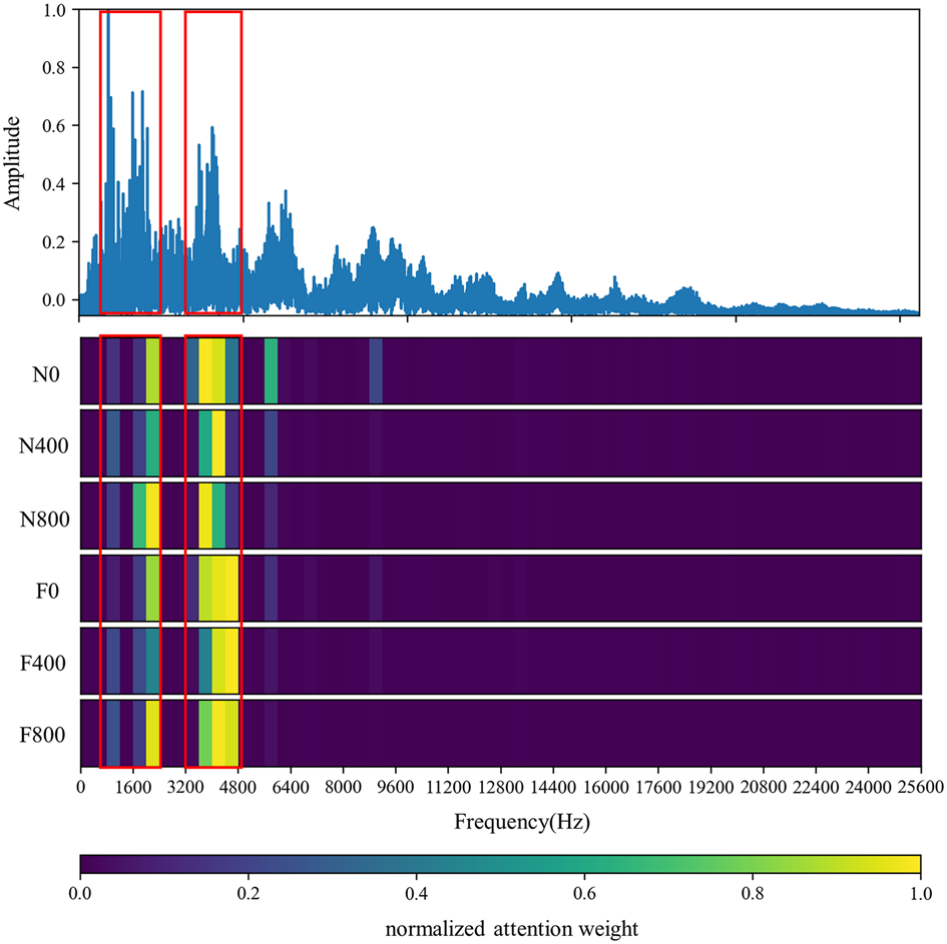
Attention for each working condition on the frequency domain.

**Figure 12:**
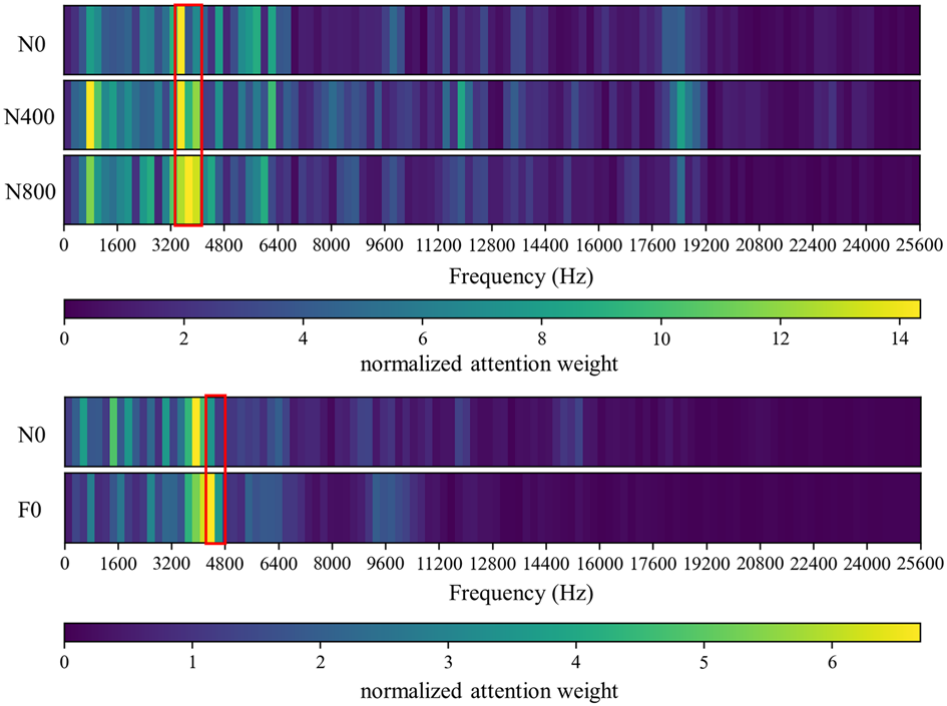
Frequency domain analysis of specific phase signal (Upper: firing; lower: valve opening).

It can be observed from Figs. 8 and 11 that the network mainly captures two peak areas within 5000 Hz in the frequency domain, namely 800–2400 Hz and 3200–4800 Hz. In case of abnormal exhaust valve clearance of diesel engine happened, the network noticed the interval near 4400–4800 Hz; with the increase of diesel engine load, the attention weight of the network to the interval of 1600–2400 Hz and 3600–4000 Hz firstly decreases and then increases. On the premise that the fault position is known, we have carried out a simple frequency domain analysis on the ignition and valve opening phase impact. As shown in Fig. 12, it is found that when there is an abnormal valve clearance, the peak value in the frequency domain moves from around 4000 Hz and falls within the range of 4400–4800 Hz; When the load increases, the spectral peak in the range of 3600–4000 Hz appears diffusion, which coincides with the attention of the network. Therefore, the method proposed in this paper has certain significance for feature interval location, but feature extraction still needs further research.

#### Envelope spectrum

In this section, the influence of the attention mechanism on the envelope spectrum is studied. Single-cylinder A4 data that is six periods samples is used as input. The number of input points is 24,576, the labeled data $N_{train} = 30$ is used for training, and the number of segments is 64.

**Figure 13:**
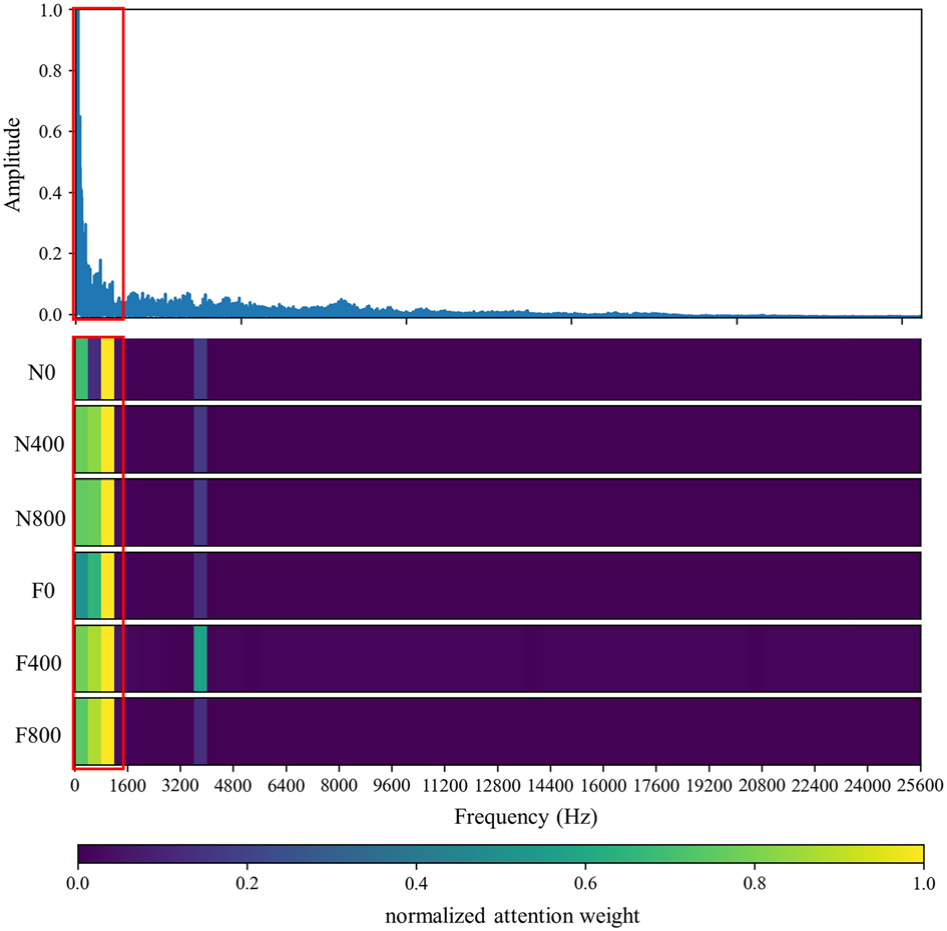
Attention for each working condition on envelope spectrum (25,600 Hz).

**Figure 14:**
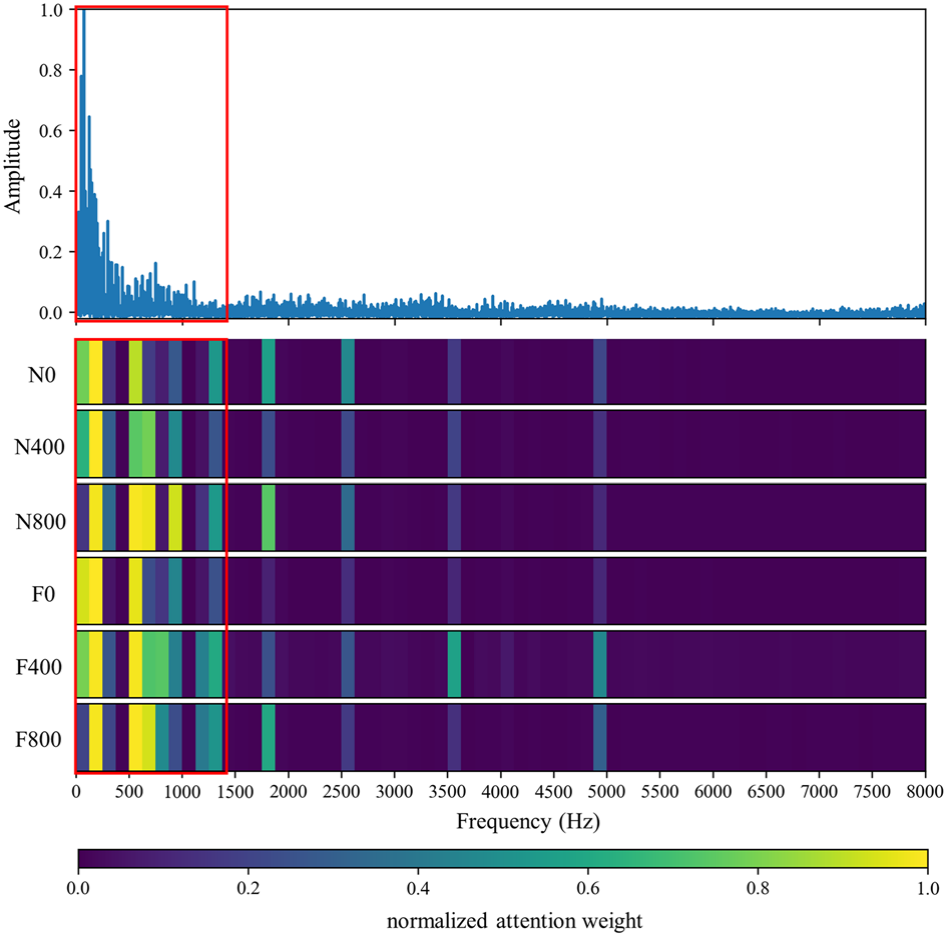
Attention for each working condition on envelope spectrum (8000 Hz).

By analyzing the envelope spectrum of 25,600 Hz in Fig. 13, it is found that the network pays attention to the data in the lower frequency region, which is consistent with the experience of artificial envelope analysis. Therefore, after further testing and analysis, the sample period is extended to 7, and 8000 Hz is intercepted as the input data. The obtained attention weight is shown in Fig. 14, and Fig. 9 is the attention visualization learned from the envelope spectrum (8000 Hz).

It can be observed that the network mainly captures the area within 1375 Hz of envelope spectrum data. When abnormal exhaust valve clearance appears, the network strengthens and pays attention to the sections near 750–875 Hz and 1125–1250 Hz. With the increase of diesel engine load, the attention of the network to the 500–1375 Hz range gradually diffuses, in which the attention to the 625–750 Hz range increases and the 0–125 Hz range decreases. The decrease in attention to the 0–125 Hz range of the envelope spectrum is probably due to the low-frequency vibration caused by the constraint unbalanced of the diesel engine under no-load conditions, which will be suppressed with the increase of load. 750–875 Hz and 1125–1250 Hz may be closely related to the fault characteristics of valve impact. 625–750 Hz of envelope spectrum and 3200–4000 Hz of spectrum signals have the same diffusion phenomenon, which is likely to be closely related to ignition impact. The generation and extraction of feature frequency need more in-depth research.

#### Results and discussion

**Figure 15:**
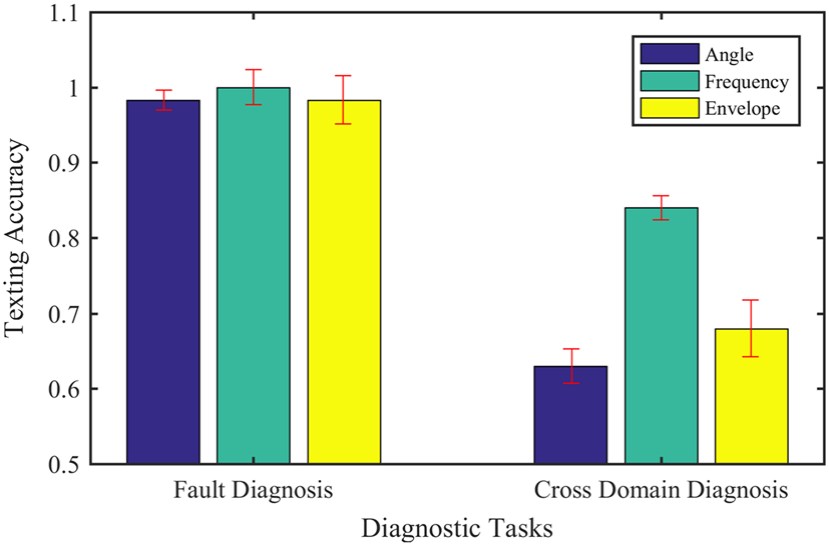
Diagnostic performance of different input methods.

This section compares the effect of different input methods on network performance and comprehensively describes the advantages and disadvantages of different input methods. The test data includes 6 working conditions shown in Table 4. 60 groups of training samples are used for each working condition, and the data contains a total of 6 cylinders from A1 to A6 to enhance the difficulty of network diagnosis. In the practical industry, due to the changes of machine working conditions and the interference of environmental noise, there are distribution differences between training and test data, resulting in a significant decline in diagnostic performance. This challenging problem is called cross-domain fault diagnosis^36^. To evaluate the performance of the model in many aspects, the network is trained with the data of 0 N m load, and the cross-domain diagnostic test is carried out under 800 N m load. To eliminate the influence of the test results caused the randomness, each group of experiments is repeated 10 times. The fault diagnosis accuracy and cross-domain diagnosis performance are shown in Fig. 15.

There is a clear relationship between angle domain signal and crankshaft angle. The interpretation of the model on the angle domain is intuitive, but its performance is poor in the cross-domain diagnosis task. Spectrum and envelope spectrum signals have better cross-domain diagnostic performance than angle domain signals. But for envelope spectrum signals, the network pays more attention to lower frequency components, which degrades its performance relative to the spectral signal. The envelope spectrum signal may lack some high-frequency characteristics and is not suitable for diesel engine fault diagnosis. For the spectrum signal, the network has a comprehensive range of concerns. At the same time, it is better than other input methods in both fault diagnosis and cross-domain diagnosis tasks. Therefore, the frequency domain signal is comprehensively selected as the input mode of the network.

### Effect of segment number

The input data needs to be segmented before it can be input into the network. When the number of segments is too small, each segment signal contains not only important feature information but also some redundant information. It is difficult for the network to produce appropriate attention weight allocation, and the performance improvement of the model is not obvious. However, when the number of segments is too large, the important features will be segmented, and the hidden features in the information will be modified, which may make the performance of the model worse. Therefore, to avoid the loss of transient information, the segmentation test adopts the combination of binary tree and trigeminal tree. At the same time, the number of segments is limited to less than 128 to avoid the excessive stacking of convolution structures, which affects the operational efficiency of the network. The number of segments is selected as follows:

27 $$n = 2^{{sort(i,i + \log_{2} 3 - 1)}} \quad (i = 1,2, \ldots ,7).$$

**Figure 16:**
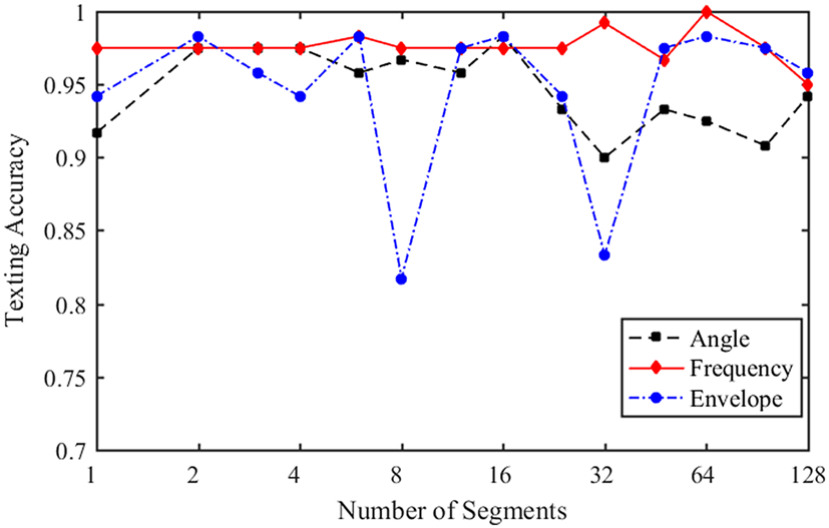
Effect of segment number on network performance.

$n$ is the number of segments and $sort$ is the sorting function. Using the data of 6 cylinders from A1 to A6, the samples of each working condition $N_{train} = 60$ are tested. Figure 16 shows the influence of segment number $n$ on the performance of the model.

As can be seen from Fig. 16, the angular domain and envelope spectrum signals are more sensitive to the number of segments $n$, $n$ of the frequency domain signal has a wider adaptive range. And the performance of the model on frequency domain signal is better than the other two. For angular domain signals, when $n = 16$, the network reaches the optimal performance. For envelope spectrum, when $n = 2,5,16,64$, the network has high classification performance, which shows that these segments are more reasonable for data cutting. However, too few segments will lead to too much information contained in each segment of the signal, which is not conducive to our further analysis of the characteristic interval. Considering comprehensively, the number of segments $n = 64$ is selected. For frequency-domain signals, when $n = 64$, the network achieves the optimal performance.

### Convolution weight setting

**Figure 17:**
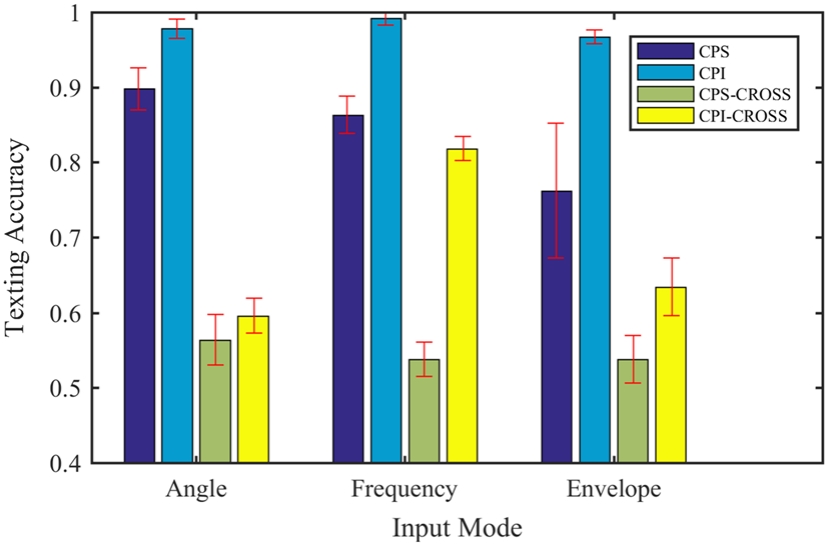
Influence of convolution weight setting on network performance.

To investigate whether the weights in each sequence convolution layer are shared or not and how they affect the performance of the proposed model, two groups of control experiments are set. All the experimental model structures are the model in Fig. 3 but do not include the attention module, to eliminate the influence of the sparse attention module. Adjust the corresponding network according to the test requirements of each group, and take the frequency domain data as the input. In the first group of experiments, the convolution parameters of each sequence are shared, which is recorded as CPS (convolution parameter sharing); In the second group of experiments, the convolution parameters of each sequence are independent and recorded as CPI (convolution parameter independence). The input data points of the two groups are 16,384. The test dataset includes 6 working conditions shown in Table 4. 60 groups of training samples are used for each working condition, and the data contains 6 cylinders from A1 to A6 to increase the diagnostic difficulty of the network. To evaluate the performance of the model in many aspects, the network is trained with the data of 0 N m load, and the cross-domain diagnostic test is carried out under 800 N m load. Cross-domain diagnostic tasks are recorded as CPS-CROSS and CPI-CROSS. To eliminate the influence of the test results caused the randomness, each group of experiments is repeated 10 times. The fault diagnosis accuracy and cross-domain diagnosis performance are shown in Fig. 17.

From Fig. 17, we can see that the CPI method is superior to the CPS method in both the accuracy of a classification and the stability of classification results in the fault diagnosis and cross-domain diagnosis tasks. In addition, the CPI method improves significantly compared with the CPS method in the face of three different input modes. It shows that the method of independent convolution parameters for each sequence has better generalization in the proposed model.

### Method comparison

To verify the performance of the proposed adaptive sparse attention network, a variety of existing intelligent diagnosis methods are compared with the proposed methods.

In the SVM method, the inputs are four time-domain features extracted from the time-domain signal: inlet valve opening impact, inlet valve closing impact, exhaust valve opening impact, and exhaust valve closing impact; In the BP method, the frequency domain signal is used as the input, and the number of nodes in each layer is 3600-60-6 respectively; In the CPI method, the network structure in “Convolution weight setting” is used, the convolution parameters of each sequence are independent, and the attention module is not included; In the Global method, the attention structure adopts the global attention mechanism; In the Local method, the attention structure adopts the local attention mechanism, which pays attention to the window area where the target sequence is located; In the proposed ASAN method, the network structure is described in Fig. 3. Each method was tested 10 times with the same dataset including 6 cylinders from A1 to A6 and 6 working conditions, and the average accuracy and loss of the test set were counted. In addition, the network is trained by the data of 0 N m load, then the cross-domain diagnosis test is performed and calculated under 800 N m load. The test results are shown in Table 5.

**Table 5:** Diagnostic performance of different methods.

Diagnostic method	Input mode	Fault diagnosis accuracy	Cross-domain diagnostic accuracy
SVM	Manual features	0.967	0.63
BP	Frequency domain	0.975	0.67
CPI		0.992	0.818
Global		1	0.84
Local		0.958	0.71
ASAN		1	0.84

**Figure 18:**
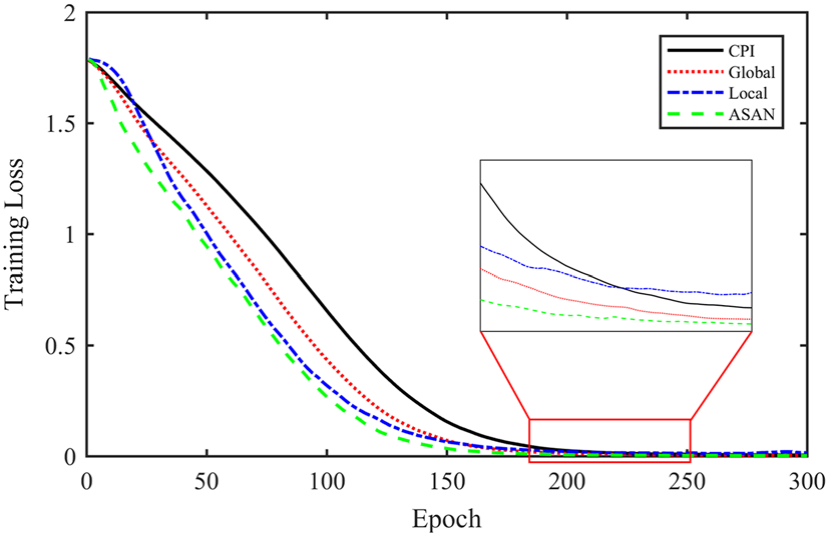
Training loss curves under different models.

Compared with the CPI method, the global method has one more layer of attention structure and increases the complexity of the algorithm. Although the convergence epochs are reduced, the training time is not shortened. The complexity of the other three attention methods is similar, the network scale is the same, and the time taken by each epoch is close. Refer to Ref.^28^ to compare and test the convergence of loss, which can reflect the training efficiency of the network to a certain extent. The results are shown in Fig. 18.

It can be seen from Table 5 and Fig. 18 that the fault diagnosis accuracy of SVM and BP methods is high, which can reach more than 95%, but the cross-domain diagnosis performance is poor. The CPI method greatly improves the cross-domain diagnosis performance of the model, but it has a certain impact on the training efficiency of the model. The Global method further improves the network performance, whose fault diagnosis accuracy reaches 100% and the cross-domain diagnosis accuracy reaches 84%. But the training time of the model has not been reduced. The Local method further speeds up the convergence speed of the model, but it will lead to the decline of fault diagnosis and cross-domain diagnosis performance. The ASAN method not only maintains the same excellent performance as the Global method in fault diagnosis and cross-domain diagnosis but also increases the efficiency of model training. Comprehensively, the proposed ASAN method is superior to other algorithms in Table 5.

**Figure 19:**
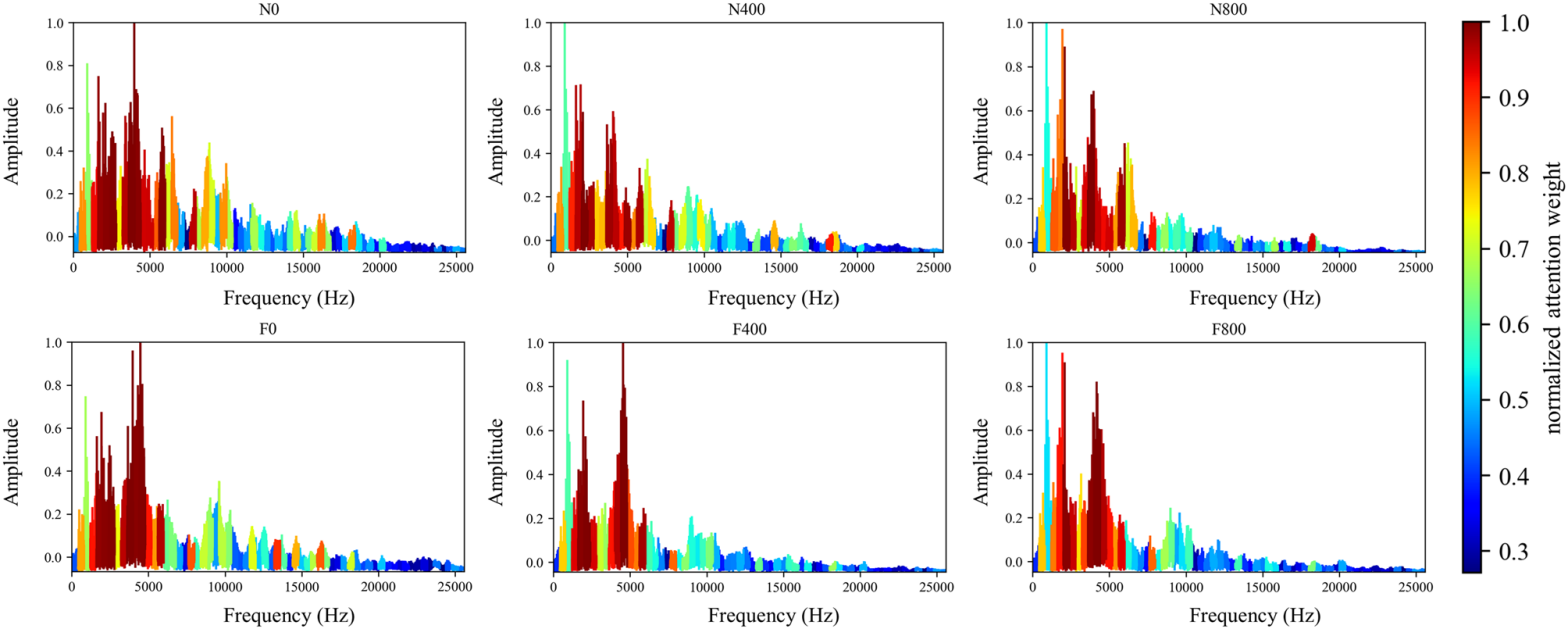
Visualization of attention weight in Global method.

**Figure 20:**
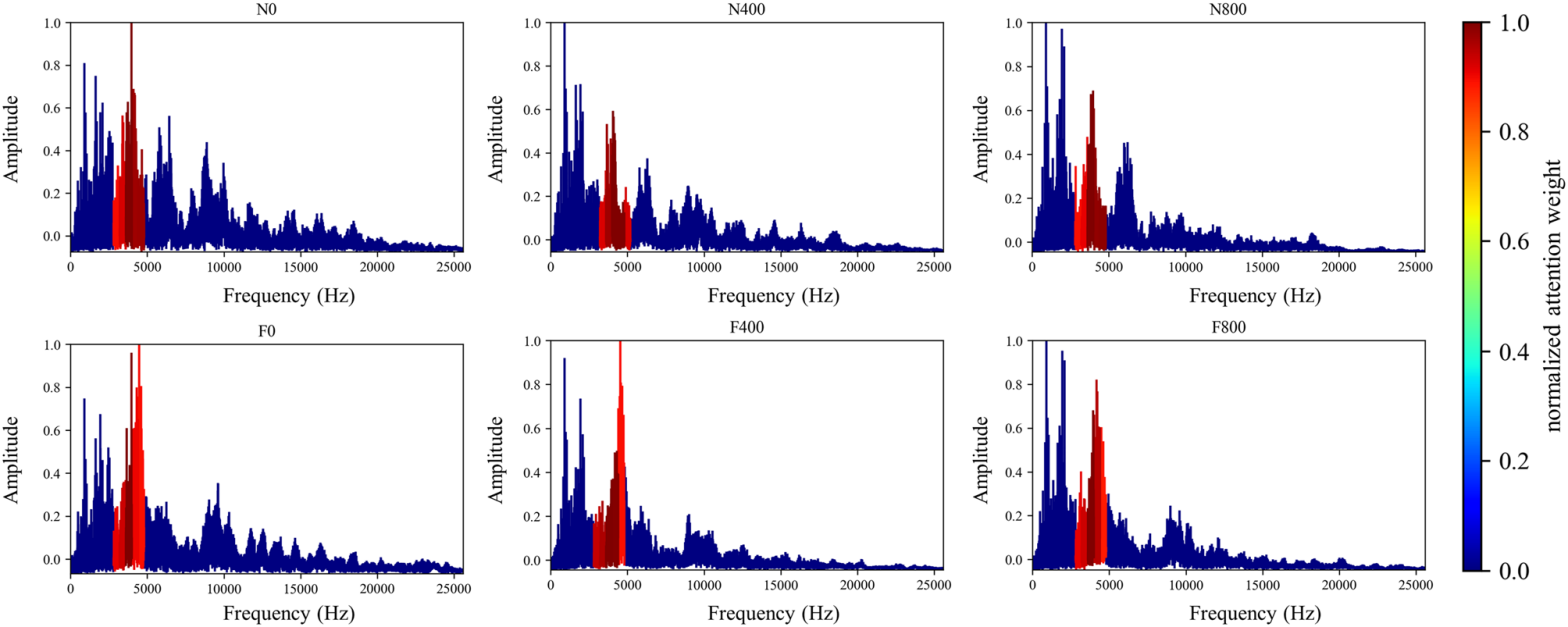
Visualization of attention weight in the Local method.

To show that the proposed algorithm has better interpretability, the attention weight of cylinder A4 samples is normalized and averaged. The visualization results of each method are shown in Figs. 8, 19 and 20, respectively. It can be seen that the Global method has a considerable degree of attention in the whole frequency band. Because of the window limitation, the Local method can only pay attention to the frequency band around 4000 Hz, which is also reflected in the three methods. The ASAN method not only retains Globals' focus but also greatly suppresses the expression of redundant sequences, which can help us better understand and diagnose fault signals.

## Conclusion

In this paper, an effective deep attention learning-based digital twin auxiliary approach for diesel engine fault diagnosis is proposed to discusses the interpretability of the network from the perspective of understanding the fault mechanism. To further improve the interpretability of the model, an adaptive soft threshold filter on the attention module is adopted to suppress redundant information dynamically in real time and combined with CNN and BiLSTM. The diesel valve fault dataset is used for experimental validation. The raw vibration angle domain data, spectrum, and envelope spectrum are used as model inputs respectively. The test shows that the output result of the model is consistent with the manual experience of fault diagnosis, and verify the effectiveness of sparse attention mechanism, especially in the spectrum. Comparisons with other approaches to verify the superiority of the proposed method.

The future work is mainly to explore the signal interval obtained through the sparse attention mechanism, further extract its internal features, and explain the causes of the features, to achieve the purpose of feedback maintenance. The collaborative updating of the model and real-time data will also be studied in the follow-up. Moreover, the proposed digital twin auxiliary approach based on ASAN needs to be further improved and verified in the actual industrial field.

## Data Availability

The datasets generated and/or analyzed during the current study are not publicly available due to the high cost of the original data collection, but are available from the corresponding author on reasonable request.
